# Lipoamide Alleviates Oxidized Fish Oil-Induced Host Inflammatory Response and Oxidative Damage in the Oviduct of Laying Hens

**DOI:** 10.3389/fvets.2022.875769

**Published:** 2022-04-04

**Authors:** Qingxiu Liu, Wenxiang Li, Jiatu Zhang, Lihong Zhao, Cheng Ji, Jianyun Zhang, Shimeng Huang, Qiugang Ma

**Affiliations:** State Key Laboratory of Animal Nutrition, College of Animal Science and Technology, China Agricultural University, Beijing, China

**Keywords:** lipoamide, oxidized fish oil, anti-oxidation, oviduct, laying hens

## Abstract

Fish oil (FO) is an important source of lipid in functional food and aquafeeds. However, the harmful effects of oxidized fish oil (OFO) on host metabolism and reproductive health are not yet clear. In addition, lipoamide (LAM) has been widely studied as an agent for alleviating various diseases associated with oxidative disruption. Therefore, in the current study, to investigate the effects of LAM in alleviating OFO-induced decline in reproductive performance and oxidative damage to the oviduct in laying hens. We constructed a 1% fresh FO model, a 1% OFO model, and a LAM model with 1% OFO (OFO + LAM) added at 100 mg/kg to explore the antioxidant effect of LAM. Herein, these results were evaluated by breeding performance, immune responses, estrogen, and antioxidant indices of serum samples, as well as the number of follicles and antioxidant parameters of oviducts. From the results, compared with the FO group, OFO significantly decreased the egg-laying rate, increased the contents of total protein (TP) and inflammatory factors [tumor necrosis factor α (TNF-α), interleukin (IL)-6, IL-8, and interferon γ (INF-γ)], and reduced the concentrations of anti-oxidation [total antioxidant (T-AOC), total superoxide dismutase (T-SOD), glutathione peroxidase (GSH-Px), glutathione (GSH), glutathione reductase (GR), catalase (CAT), and hydroxyl radical scavenging activity (HRSA)] in serum samples, as well as reduced the levels of anti-oxidation indexes in oviduct tissues (*p* < 0.05). Of note, the supplementation of LAM could significantly increase the laying performance, improve the levels of serum immunoglobulins (IgA, IgG, and IgM), serum estrogen [progesterone (P) and estradiol (E2)], and serum antioxidant parameters (T-AOC, T-SOD, GSH-Px, GSH, GR, CAT, and HRSA) and decrease the concentrations of serum inflammatory cytokines (TNF-α, IL-6, IL-8, and INF-γ) in laying hens following OFO administration (*p* < 0.05). In addition, LAM could dramatically increase the contents of antioxidant factors (*p* < 0.05) in oviducts and enhance the secretion capacity of the uterine part. Taken together, OFO caused host metabolic dysfunction, oxidative damage, uterine morphological abnormalities, and alterations of ovarian function. These results suggested that LAM administration could alleviate host metabolic dysfunctions and inflammatory damage, and then ameliorate oxidative damage in the oviduct induced by OFO, ultimately improving reproductive function.

## Introduction

During layer breeding, problems, such as a rapid decline in the egg production rate of old laying hens and low quality of late commercial eggs, are limiting the production efficiency. It is generally accepted that the decline in fertility is mainly affected by the aging of the reproductive organs (1). Most of the health problems related to aging are associated with excessive oxidative stress and inflammation in the body. It is hypothesized that the main reasons for the decline in egg production rate in old laying hens are the oxidative damage and the inflammatory response to the oviductal mucosa induced by the physiological stress of long-term high production, oxidized fat in the diet, and aflatoxin (AFB) accumulation poisoning (2). A study that lasted 23 years showed that losses due to salpingitis in old laying hens accounted for 1–8% of total losses on the farm (3). This could mean that the health of oviduct greatly limits the efficiency of the utilization of old laying hens. Therefore, alleviating oviductal oxidative stress, reducing incidence of salpingitis, and maintaining oviduct health may be important directions to improve egg production in old laying hens.

As we all know, fish oil (FO) has a unique biological function of promoting the metabolism of saturated fatty acids *in vivo*. However, due to its high degree of unsaturation, FO is highly susceptible to oxidation, and the harmful effects of oxidized fish oil (OFO) on animal production have been widely reported. Numerous studies have shown that feeding OFO to experimental animals can result in the production of excessive reactive oxygen species (ROS), which can lead to the production of MDA and consumption of SOD, then induce the formation of oxidative stress (4–7). The consumption of OFO by animals will have a negative impact on the growth performance and antioxidant capacity of the animal body, reduce the resistance of animals to diseases, and reduce the quality of animal products.

Lipoic acid (LA), a conditionally essential nutrient that slows or repairs oxidative damage and exhibits strong antioxidant activity (8), is considered an effective agent in the prevention or treatment of certain diseases of aging (9). In a trial of polycystic ovary syndrome women, LA was found to significantly increase antioxidant levels in reproductive organs and improve reproductive function (10). In poultry, several studies have found that LA significantly increased the activity of the antioxidant enzyme and total antioxidant (T-AOC) in serum and liver samples, as well as reduced the level of lipid peroxidation *in vivo* under normal dietary conditions in broilers (11–13). Lipoamide (LAM) is the most important neutral amide of LA, and these two compounds have similar structures and biological capacities (14, 15). Studies have reported that LAM is an antioxidant *in vitro* (16), and LAM had a greater antioxidant effect than LA (17, 18). Hou et al. found that LAM could resist oxidative stress-mediated neuronal cell damage. Besides at the same concentration, the antioxidant effect of LM was significantly better than LA (19). Regarding the protective effect of LM better than LA, and it may be since LM has a higher lipid solubility, so its ability to adapt to the body environment exceeds that of LA (20, 21).

Oxidative stress is the main cause for the degeneration of oviduct function and salpingitis in laying hens (22). LAM, as a powerful antioxidant, may have a great potential to inhibit oxidative damage. However, whether LAM has the effect to alleviate oxidative damage in the oviduct is not yet known. Therefore, our study was conducted to construct a model of OFO-induced stress in old laying hens and then explored the adverse effects of LAM mitigation of oxidative stress to develop a feed additive to alleviate oviduct inflammation and oxidation in laying hens.

## Materials and Methods

All protocols related to animal use in this study were approved by the Institutional Animal Care and Use Committee of the Chinese Agricultural University.

### Animal Husbandry and Experiment Design

Two experiments were conducted separately in this study. Experiment 1 was designed to investigate the effect of dietary supplementation of LAM on reproductive performance indicators, such as egg-laying rate, egg weight, egg production, and the feed-to-egg ratio of old laying hens. First, a total of 60 commercial laying hens of the Peking Red strain (Yukou Poultry Co., Ltd. of Beijing, China) at the age of 106 weeks with a similar laying performance were randomly divided into two treatments control group (CON) and added 100 mg/kg LAM group (LAM). Each of the groups consisted of 15 replicates (two laying hens per replicate) in 15 different cages (two birds per cage). Cages (H 45 × W 45 × D 45 cm) were equipped with one nipple drinker and an exterior feed trough that expanded the length of the cage. Hens were raised in an enclosed, ventilated, and conventional house with 16 h-light and 55% relative humidity on average. Feed and water were provided *ad libitum* during the entire experimental period. For 2 weeks of pre-feeding, all treatments were fed a corn-soybean meal diet for late laying hens. From 108 weeks (week 1 of the experiment), each treatment was fed the corresponding diet and started a 16-week observation period until the end of 123 weeks of age (week 16 of the experiment). The weekly egg-laying rate, egg weight, and feed intake were recorded for 16 weeks, and egg production and feed-to-egg ratio were calculated.

Experiment 2 was designed to investigate the effect of LAM on reproductive performance, blood immune, inflammatory and hormonal indexes, and antioxidant indices of serum and uterine part of old laying hens under oxidative stress. From 124 weeks (week 17 of the experiment), the 30 hens in the CON group in experiment 1 were divided into two groups and supplemented with 1% fresh FO and 1% OFO, respectively. The peroxide values of fresh FO and OFO were 3.65 and 184.54 meq/kg, respectively. Each treatment had 15 replicates with one bird per replicate, placed in the same cage. In addition, 15 laying hens in the LAM group in experiment 1 were randomly selected and supplemented with 1% OFO (OFO + LAM). At the end of 127 weeks of age (week 20 of the experiment), the reproductive performance of the three treatment groups was recorded. The basal corn-soybean meal diet was formulated to meet the requirements of Peking Red laying hens (NYT33-2004) (Supplementary Table 1). During the experiment period, the hens were fed two times a day.

### Sample Collection and Treatment

At the end of the experimental period (127th week), one bird close to average weight was selected for each replicate. The selected birds were first weighed. Then, wing venous blood was collected, and serum was obtained by the centrifugation of a respective blood sample at 3,000 r/min for 15 min at 4°C and stored at −80°C until further analysis. After blood collection, the birds were sacrificed and dissected. The uterus was collected by simultaneously washing with cold sterile PBS to remove the attached impurity and gently scraped. Uterine samples were diluted with the nine-time volumes of sterile ice-cold normal saline (0.9%) based on the sample weight and then homogenized using a hand-held glass homogenizer. The tissue supernatants were collected by centrifuging at 3,500 × g for 10 min at 4°C, and the concentration of protein was determined by a BCA protein assay kit according to the manufacturer's instruction (Pierce, Rockford, IL) and stored at −80°C for further study. Finally, the ovaries and oviducts were separated, photographed, weighed, the length of the oviducts was measured, the number of different forms of follicles was counted, then the data obtained were recorded.

### Serum Biochemical, Immune, Inflammatory, and Hormone Parameters

The levels of total protein (TP), albumin (ALB), immunoglobulin A (IgA), immunoglobulin G (IgG), immunoglobulin M (IgM), tumor necrosis factor α (TNF-α), interleukin 1β (IL-1β), interleukin 6 (IL-6), and interferon γ (IFN-γ) in serum were measured with kits (Nanjing Jiancheng Bioengineering Institute, Nanjing, China). The level of estradiol (E2) and progesterone (P) in serum were determined with commercial radioimmunoassay (RIA) kits in accordance with the manufacturer's instructions (Nanjing Jiancheng Bioengineering Institute, Nanjing, China).

### Antioxidant Assay of Serum and Uterus

The serum and uterus antioxidant indices were detected using commercial kits (Nanjing Jiancheng Bioengineering Institute, Nanjing, China). The concentrations of total antioxidant (T-AOC), total superoxide dismutase (T-SOD), catalase (CAT), glutathione reductase (GR), glutathione peroxidase (GSH-Px), glutathione (GSH), hydroxyl radical scavenging activity (HRSA), and malondialdehyde (MDA) in serum and uterine part were measured in accordance with the manufacturer's instructions. The results were normalized to protein concentration in each uterine homogenate.

### Hematoxylin-Eosin (H & E) Staining and Histopathology Analyses

Different portions of the uterine were collected to assess histopathological damage. Then, the isolated uterine was flushed with PBS, and the uterine part was separated into two parts. One part was snap-frozen in liquid nitrogen, and then stored at −80°C in a refrigerator for subsequent analysis. Another tissue portion was fixed in 4% paraformaldehyde, included in paraffin, cut into micro-sections of 5 μm, mounted on glass slides, and finally stained with hematoxylin (Solarbio, Beijing, China) and eosin Y solution (Solarbio, Beijing, China). Then, hematoxylin and eosin (H&E) stained paraffin sections were viewed under bright fled on a Zeiss Axio Imager microscope as outlined previously. Microscopic intestinal damage was observed in images using the measurement tool on Case Viewer software at ×200 magnification.

### Statistical Analysis

In experiment one, the original reproductive performance data were analyzed by the unpaired *t*-test using SPSS 25.0 (SPSS Inc., Chicago, IL, USA). Numerical results are expressed as mean, with *p* < 0.05 being considered significant.

In experiment 2, the original reproductive performance, immune, inflammatory and hormone factors, and antioxidant indexes were conducted using one-way ANOVA available with the SPSS 25.0 (SPSS Inc., Chicago, IL, USA). The treatment means were separated by Duncan's multiple range tests at *p* < 0.05 significance levels.

## Results

### Effect of LAM on Laying Performance of Laying Hens Under Normal Feed

As shown in experiment 1 (Table 1), compared with the control group, there was no significant effect of dietary supplementation with LAM on egg-laying rate, egg weight, egg production, feed intake, laying hen weight, and feed/egg ratio in the experiment of 1–16 weeks (*p* > 0.05). However, there was a positive effect trend of LAM on the reproductive performance of older laying hens under normal feed.

**Table 1:** The effect of lipoamide (LAM) on the reproduction performance of old laying hens at 108–123 weeks.

**Item**	**Egg laying rate/%**	**Egg weight/g**	**Egg production/g/d**	**Feed intake/g**	**Laying hens weight (the final BW)/kg**	**Feed/egg ratio**
CON	77.99	65.64	50.88	117.0	1.712	2.438
LAM	80.22	66.06	52.09	117.5	1.765	2.259
SEM	3.096	2.216	2.048	1.030	0.061	0.1241
*P*-value	0.480	0.851	0.293	0.603	0.396	0.165

### Effect of LAM on Laying Performance of Laying Hens Under Oxidative Stress

In experiment 2 (Table 2), compared with the FO group, the OFO group had a significantly lower egg-laying rate (*p* < 0.05) and a decreasing trend in egg production (*p* > 0.05). There were no significant differences in egg weight, feed intake, laying hen weight, and feed-to-egg ratio (*p* > 0.05). On the other hand, compared with the OFO group, the egg-laying rate and egg production in the OFO + LAM group were significantly increased (*p* < 0.05), but there were no significant changes in the egg weight, feed intake, laying hen weight, and feed/egg ratio (*p* > 0.05).

**Table 2:** The effect of fish oil, oxidized fish oil, and oxidized fish oil with LAM on the reproductive performance of old laying hens.

**Items**	**Egg laying rate/%**	**Egg weight/g**	**Egg production/g/ d**	**Feed intake/g**	**Laying hens weight (the final BW)/kg**	**Feed/egg ratio**
FO	73.85^b^	64.67	47.71^ab^	114.33	1.69	2.47
OFO	63.93^a^	67.16	43.14^a^	116.41	1.73	3.23
OFO + LAM	78.77^c^	68.16	53.45^b^	117.58	1.69	2.23
SEM	3.597	1.843	2.604	1.661	0.052	0.332
*P*-Value	0.022	0.399	0.031	0.387	0.804	0.108

a, b, c *Means with different superscripts within a column differ significantly (p < 0.05)*.

### Effect of LAM on Serum Biochemical Parameters, Immunoglobulin Levels, Inflammatory Cytokines, and Antioxidant Capacity of Laying Hens Under Oxidative Stress

**Figure 1:**
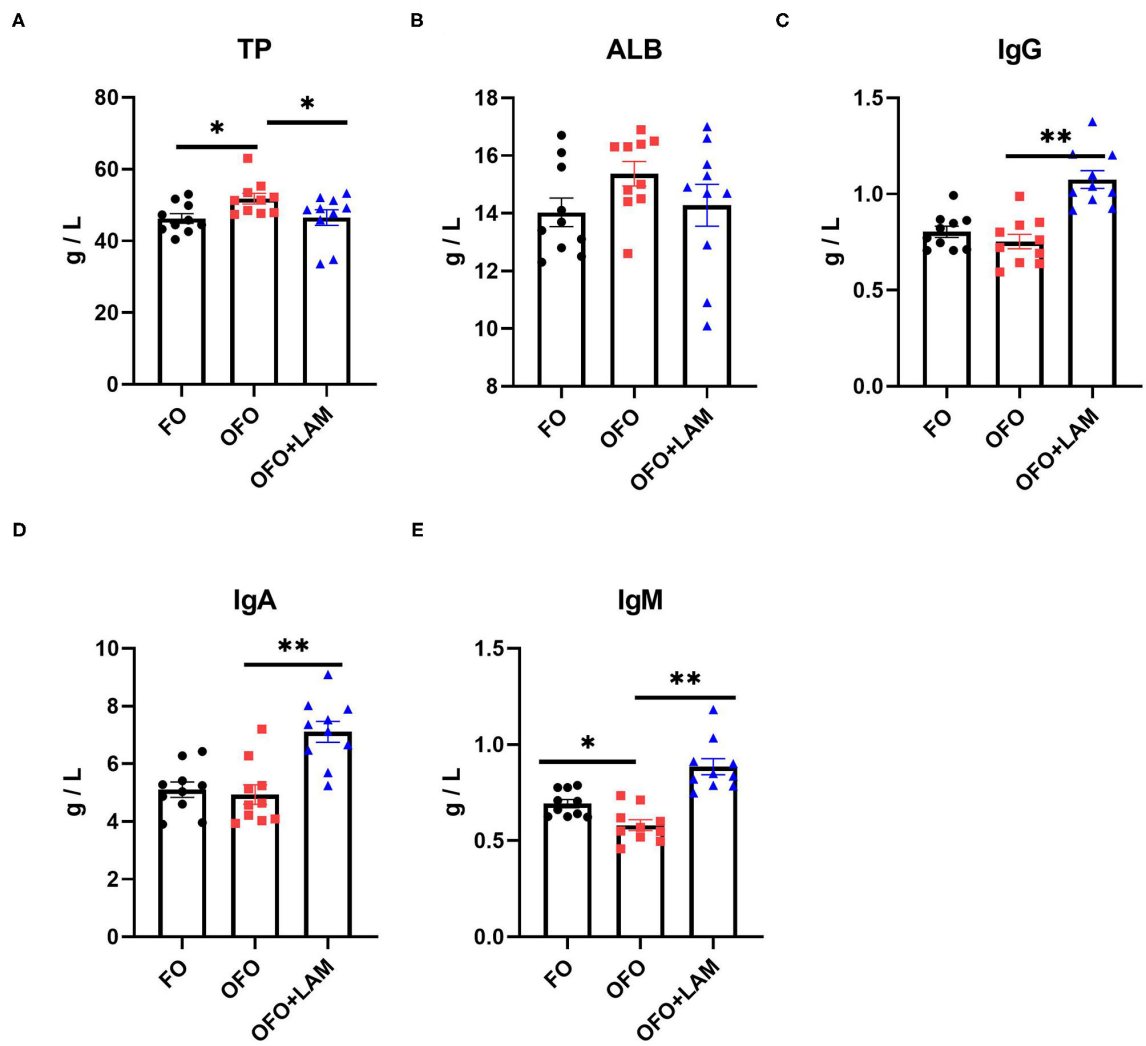
The effect of fish oil (FO), oxidized fish oil (OFO), and OFO with lipoamide (LAM) on serum biochemical and immunological indicators. **(A)** Content of total protein (TP) in FO, OFO, and OFO + LAM. **(B)** Content of albumin (ALB) in FO, OFO, and OFO + LAM. **(C)** Content of immunoglobulin A (IgA) in FO, OFO, and OFO + LAM. **(D)** Content of immunoglobulin G (IgG) in FO, OFO, and OFO + LAM. **(E)** Content of immunoglobulin M (IgM) in FO, OFO, and OFO + LAM. **p < 0.01 and *p < 0.05 indicate significant differences between two groups.

As shown in Figure 1, compared with the FO group, the level of TP was significantly increased and the content of IgM was significantly decreased in the OFO group (*p* < 0.05), but there were no significant differences in the content of ALB, IgA, and IgG (*p* > 0.05). In OFO + LAM group, the contents of IgG, IgA, and IgM were significantly higher than that in the OFO group (*p* < 0.05).

**Figure 2:**
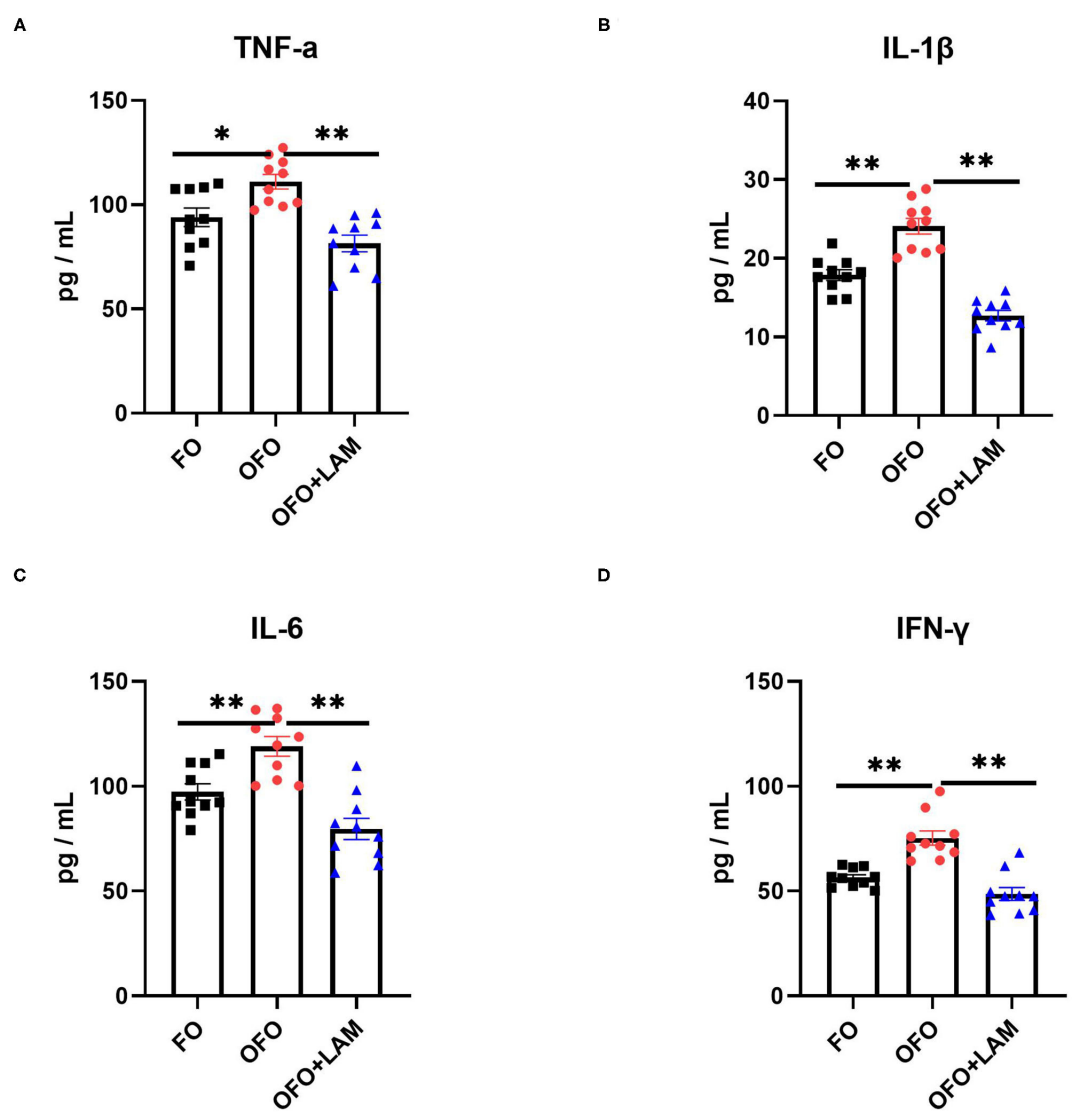
The effect of FO, OFO, and OFO with LAM on serum inflammatory cytokines. **(A)** Content of tumor necrosis factor α (TNF-α) in FO, OFO, and OFO + LAM. **(B)** Content of interleukin 1β (IL-1β) in FO, OFO, and OFO + LAM. **(C)** Content of interleukin 6 (IL-6) in FO, OFO, and OFO + LAM. **(D)** Content of interferon γ (IFN-γ) in FO, OFO, and OFO + LAM. **p < 0.01 and *p < 0.05 indicate significant differences between two groups.

**Figure 3:**
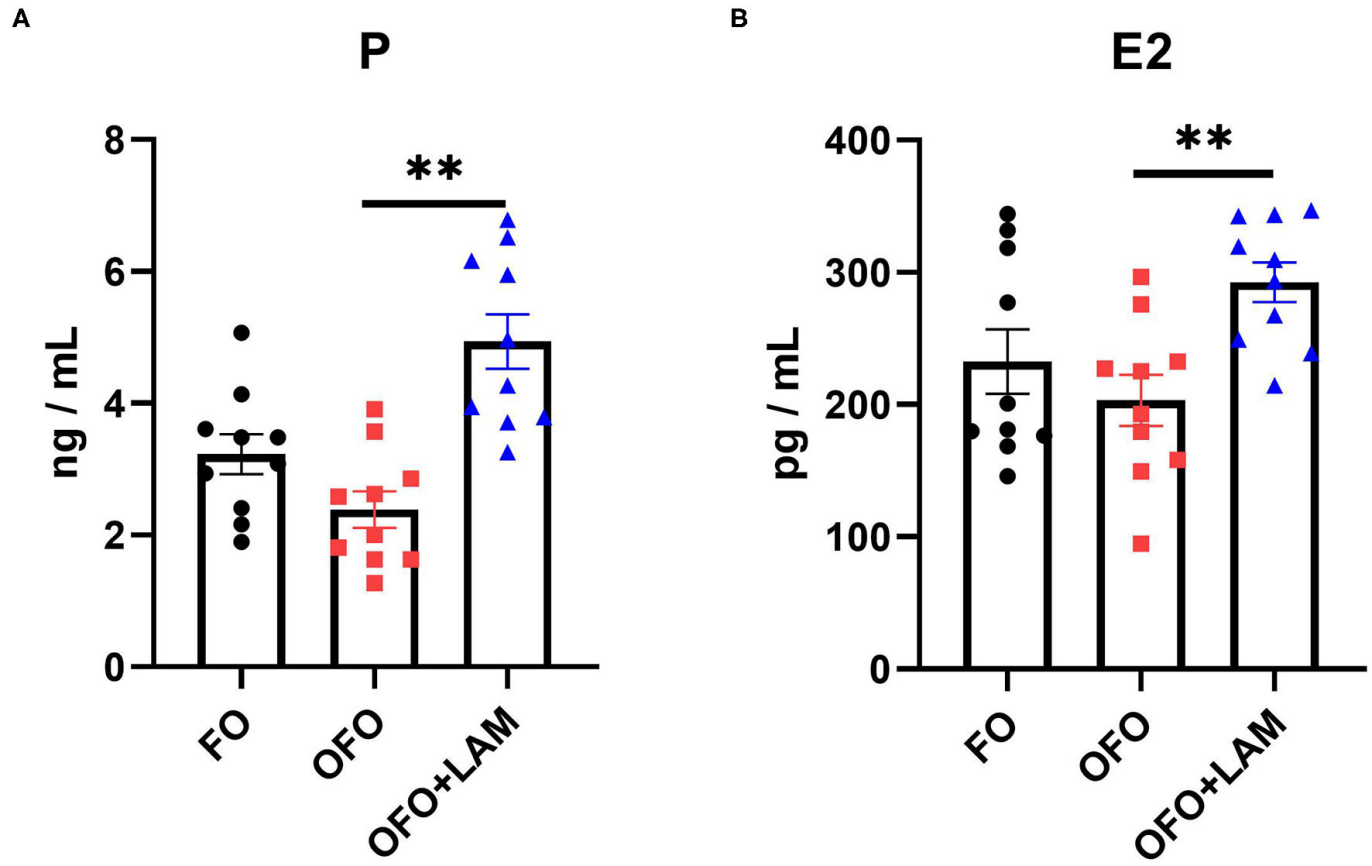
The effect of FO, OFO, and OFO with LAM on serum hormone levels. **(A)** The level of progesterone in FO, OFO, and OFO + LAM; **(B)** the level of estradiol in FO, OFO, and OFO + LAM. **p < 0.01 indicate significant differences between the two groups.

**Figure 4:**
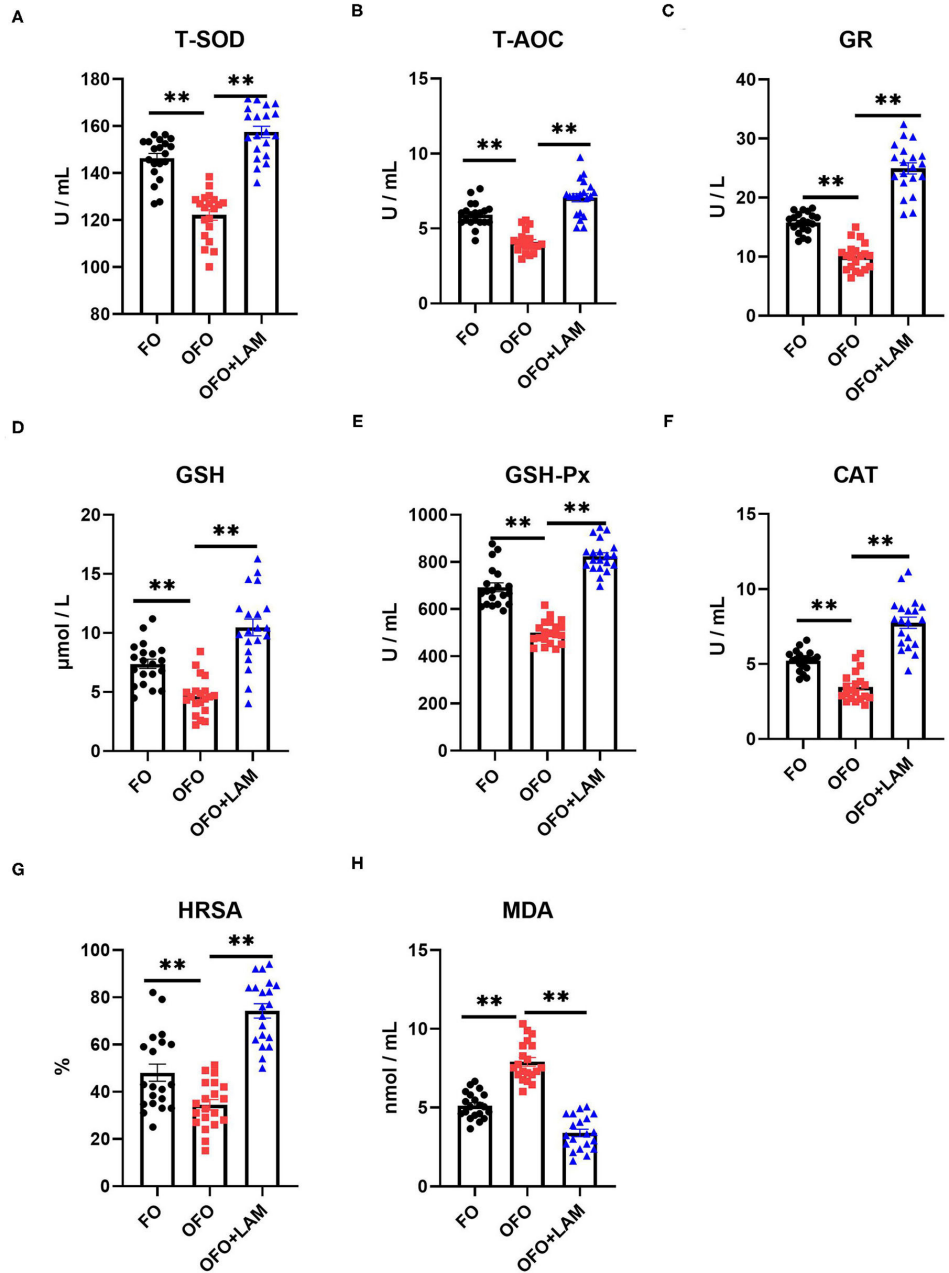
The effect of FO, OFO, and OFO with LAM on serum oxidative stress parameters. **(A)** Content of total superoxide dismutase (T-SOD) in FO, OFO, and OFO + LAM. **(B)** Content of total antioxidant (T-AOC) in FO, OFO, and OFO + LAM. **(C)** Content of glutathione reductase (GR) in FO, OFO, and OFO + LAM. **(D)** Content of glutathione (GSH) in FO, OFO, and OFO + LAM. **(E)** Content of glutathione peroxidase (GSH-Px) in FO, OFO, and OFO + LAM. **(F)** Content of catalase (CAT) in FO, OFO, and OFO + LAM. **(G)** Content of hydroxyl radical scavenging activity (HRSA) in FO, OFO, and OFO + LAM. **(H)** Content of malondialdehyde (MDA) in FO, OFO, and OFO + LAM. **p < 0.01 indicate significant differences between the two groups.

As shown in Figure 2, compared with the FO group, the contents of TNF-α, IL-1β, IL-6, and IFN-γ were significantly increased in the OFO group (*p* < 0.05). In OFO + LAM group, the contents of TNF-α, IL-1β, IL-6, and IFN-γ were extremely significantly reduced than that in OFO group (*p* < 0.05). Additionally, (Figure 3) there was no significant difference in P and E2 levels in the OFO group compared with the FO group, but there was a trend toward a decrease in both groups (*p* > 0.05). Compared with the OFO group, the levels of P and E2 were significantly increased in the OFO + LAM group (*p* < 0.05). Importantly (Figure 4), compared with the FO group, the contents of T-AOC, T-SOD, GSH-Px, GSH, GR, CAT, and HRSA were significantly decreased, and the level of MDA was significantly increased in the OFO group (*p* < 0.05). On the other hand, the levels of T-AOC, T-SOD, GSH-Px, GSH, GR, CAT, and HRSA were significantly higher in the OFO + LAM group than in the OFO group, while MDA was significantly lower than in the OFO group (*p* < 0.05).

### Effect of LAM on Uterine Part Morphology and Antioxidant Capacity of Laying Hens Under Oxidative Stress

**Figure 5:**
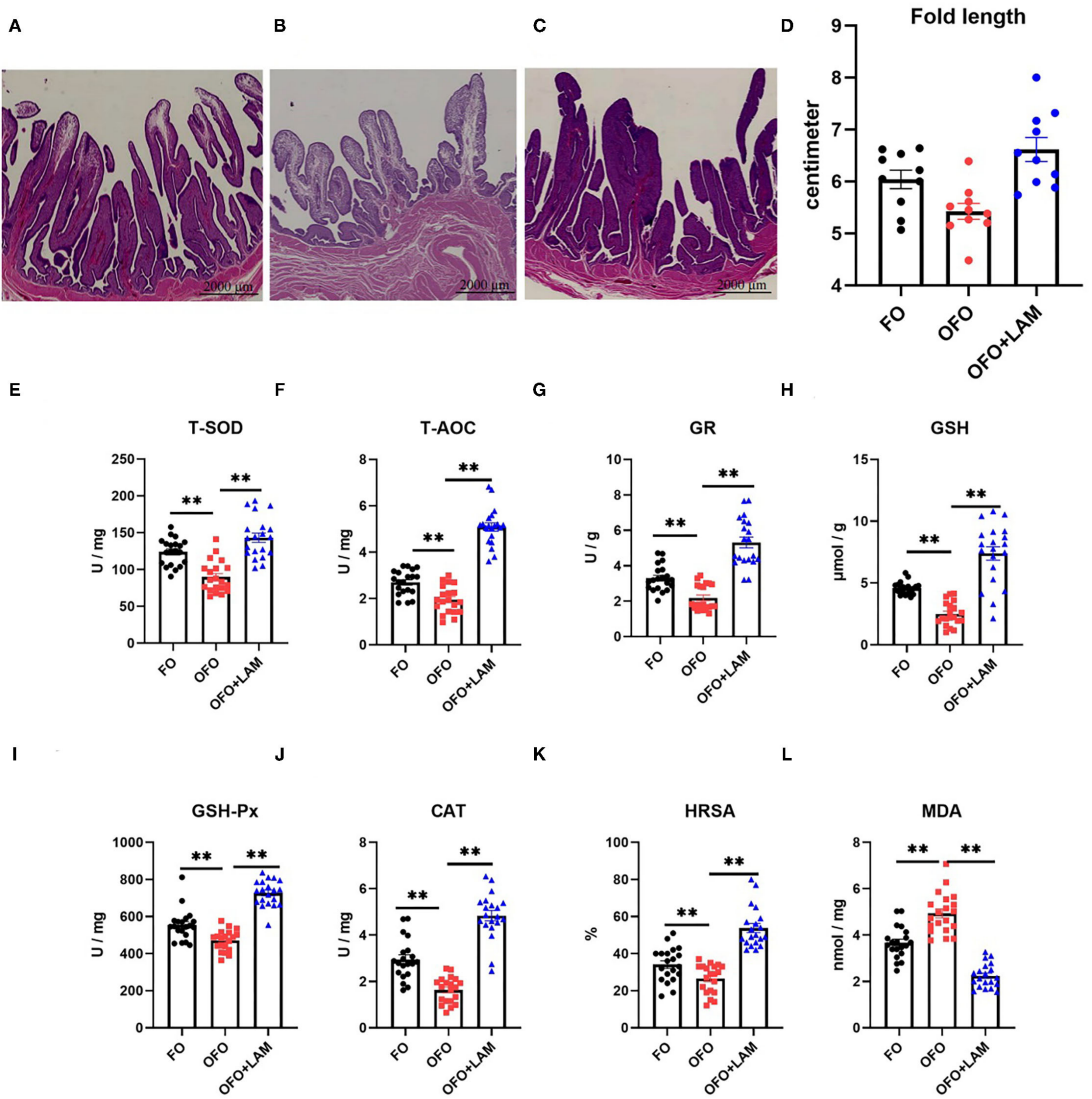
The effect of FO, OFO, and OFO with LAM on uterine part structure and anti-oxidation. **(A)** FO group; **(B)** OFO group; **(C)** OFO + LAM group; **(D)** the fold length of uterine part in FO, OFO, and OFO+LAM group. Graphs were observed at 0.5× , the size unit of the photograph is 2,000 μm. **(E)** Content of T-SOD in FO, OFO, and OFO + LAM. **(F)** Content of T-AOC in FO, OFO, and OFO + LAM. **(G)** Content of GR in FO, OFO, and OFO + LAM. **(H)** Content of GSH in FO, OFO, and OFO + LAM. **(I)** Content of GSH-Px in FO, OFO, and OFO + LAM. **(J)** Content of CAT in FO, OFO, and OFO + LAM. **(K)** Content of HRSA in FO, OFO, and OFO + LAM. **(L)** Content of MDA in FO, OFO, and OFO + LAM. **p < 0.01 and *p < 0.05 indicate significant differences between two groups.

Tubular glands in FO group are sparsely packed into the head of mucosal folds, and tightly packed into the root of mucosal folds (Figure 5A). In OFO group, tubular glands are sparsely packed into the mucosal folds (Figure 5B). Compared with the OFO group, tubular glands are tightly packed into the mucosal folds in the OFO + LAM group, and the glands are lined with cells filled with fine eosinophilic granules (Figure 5C). The mean fold lengths in the different treatments were measured (Figure 5D). The mean fold lengths in the OFO group were shorter than those in the FO group, but there was no significant difference between the two groups (*p* > 0.05). However, the mean fold length of OFO + LAM group was significantly longer than that of OFO group (*p* < 0.05).

The effect of FO, OFO, and OFO + LAM on uterine part antioxidant capacity is shown in Figures 5E–L. Compared with the FO group, the contents of T-AOC, T-SOD, GSH-Px, GSH, GR, CAT, and HRSA were all significantly decreased, and the MDA content was significantly increased in the OFO group (*p* < 0.05). Compared with the OFO group, the contents of T-AOC, T-SOD, GSH-Px, GSH, GR, CAT, and HRSA were all significantly increased, and the MDA content was significantly decreased in OFO + LAM group (*p* < 0.05).

### Effect of LAM on Ovary and Oviduct of Aged Laying Hens Under Oxidative Stress

**Figure 6:**
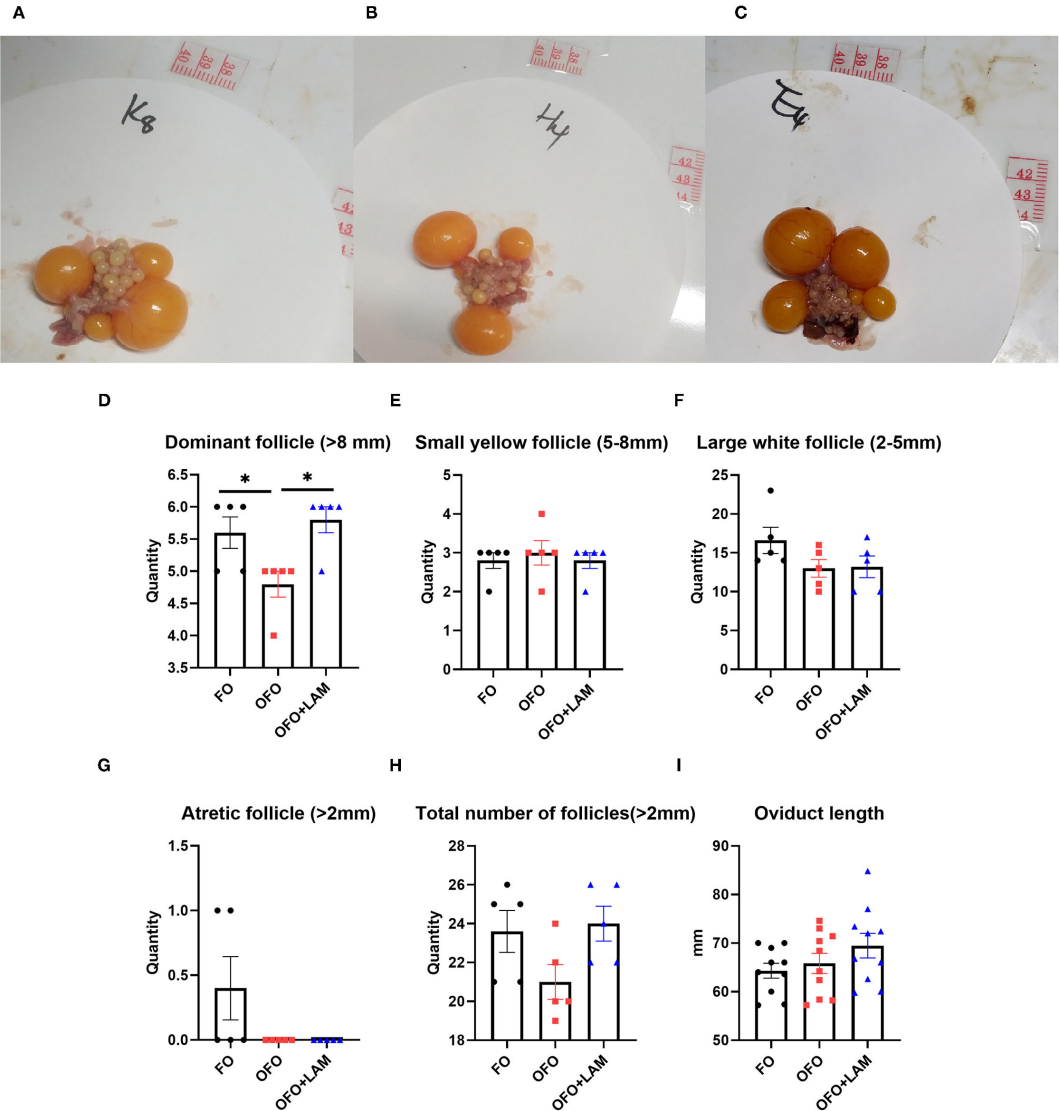
Effect of FO, OFO, and OFO with LAM on ovary and oviduct of old laying hen. Ovary figure in FO **(A)**, OFO **(B)**, and OFO + LAM **(C)**. **(D)** Number of dominant follicle (> 8 mm) in FO, OFO, and OFO + LAM. **(E)** Number of small yellow follicle (5–8 mm) in FO, OFO, and OFO + LAM. **(F)** Number of large white follicle (2–5 mm) in FO, OFO, and OFO + LAM. **(G)** Number of atretic follicle (>2 mm) in FO, OFO, and OFO + LAM. **(H)** Total number of follicle (>2 mm) in FO, OFO, and OFO + LAM. **(I)** Oviduct length in FO, OFO, and OFO + LAM. *p < 0.05 indicates significant differences between the two groups.

In the terms of the number of follicles (Figures 6A–H), the number of dominant follicles in the OFO group is significantly decreased compared with the FO group (*p* < 0.05). Compared with the OFO group, the number of dominant follicle (*P* < 0.05) and total number of follicle (0.05 < *P* < 0.1) increased in the OFO + LAM group, while there were no significant changes in another follicle number (*p* > 0.05). As shown in Figure 6I, there was no significant difference in oviduct length among the three groups, but the oviduct length in the OFO + LAM group was increased to some extent compared with the OFO group (*p* > 0.05).

## Discussion

The effects of OFO on animal performance have been studied by several authors, and most studies have shown that OFO impaired animal performance and reduces the digestive and absorption utilization of nutrients (5, 23). Especially, in the terms of oxidative stress, the addition of 15% OFO to diets produced oxidative damage in a variety of animal organisms, mainly through the release of ROS, excessive production of MDA, reduction of antioxidant enzymes, such as SOD, and oxidative stress in multiple organs and tissues (4–7). Oxidative stress is defined as an imbalance between the production of free radicals and their elimination. This imbalance leads to the damage of important biomolecules and cells, with potential impact on the whole organism (24). Song et al. (25) found that dietary 6% OFO inhibited growth performance and destroyed intestinal integrity in fish. The current study has corroborated with the previously reported that diets supplemented with OFO increased the content of inflammation factors (TNF-α, IL-1β, IL-6, and IFN-γ), antioxidant enzymes (T-AOC, T-SOD, GSH-Px, GSH, GR, CAT, and HRSA), reduced MDA content and eventually led to a decrease in the egg-laying rate of old laying hens compared with fresh FO. It also represents that OFO can successfully induce oxidative stress models in old laying hens.

Lipoic acid is a sulfur-containing fatty acid, which can rapidly scavenge oxygen free radicals when it enters the body and has a good antioxidant function. LAM, a carboxylic acid derivative of LA, is gradually becoming an alternative to LA because it has better biomedical functions and is more suitable for use in humans than LA (21). Many observations have shown that LAM is a more potent antioxidant than LA in the terms of oxidative damage (15), such as in the protection of macrophage-like cells from oxidative injury (26), and neuronal cells from glutamate-induced cell injury (27). However, it is not clear whether the addition of LAM to the diet will have a negative effect on laying hens. Therefore, we added LAM-containing feed to old laying hens which were in normal production at first, and found that LAM did not negatively affect egg production and reproduction, and even had some beneficial functions.

Egg production rate is an important indicator of egg production efficiency. In the later stages of egg production, a decrease in the egg production rate can affect the economic efficiency of the farmer. Nutritional regulation can improve the egg production rate and extend the egg-laying period of laying hens. Numerous studies have found that dietary supplementation with antioxidants can improve egg production in laying hens. For example, Kothari et al. found that the dietary supplementation of fermented pine needle extract increased egg production and feed intake during the entire experimental period (28). Liu et al. (29) revealed that the egg-laying rate had a quadratic correlation with the level of quercetin and was maximized by the supplementation level of 0.2 g/kg of diet. As a strong antioxidant, we believe that LAM has a beneficial effect on egg production performance in laying hens, and similarly our experiments have demonstrated that the dietary supplementation of LAM significantly improved egg-laying rate and egg production in old laying hens. In addition, a study found that addition of LA to the diet increased egg-laying rate, but the difference was not significant (30). Taken together, the results suggested that LAM may be more effective than LA in poultry production.

Egg production is the main factor limiting the efficiency of egg utilization during the late egg-laying period, while ovary and oviduct aging is the main reason for the decline in egg production (31, 32). Oxidative stress is deemed to be one of the dominant mechanisms underlying ovarian and oviduct aging (30). A stress experiment in rats found that chronic stress could increase MDA concentration and elicit an irreversible decrease in antioxidant defense in the oviduct (33). Antioxidants have been found to be effective in alleviating oxidative damage to the oviduct. Astaxanthin has an antioxidative effect on bovine oviduct epithelial cells due to the induction of antioxidant genes (34). In the present study, addition of LAM to the diet significantly increased the antioxidant indexes of the oviduct uterine part and serum. Consistent with our results, several studies have indicated that LA could improve antioxidant and immune function in laying hens, improve egg quality, improve meat quality (13, 35), and enhance antioxidant levels in puppies and rats (36, 37). In addition, as a nutritional supplement, LA has been reported to protect against oxidative stress-induced diabetic neuropathy and insulin resistance in humans (38). In humans, LAM may have a better antioxidant effect on certain diseases.

It is well known that the immune system is a multifaceted and complex network that protects the host from aggression. An inflammatory response is an important component of the innate immune system response to a variety of challenges (39). Furthermore, inflammatory response as one of the direct responses induced by oxidative stress (40, 41). Increasing evidence had proposed that continuous oxidative stress leads to chronic inflammation, which is one of the main causes of chronic diseases (42). A study found that as a coenzyme of mitochondria, LA can alleviate the loss of mitochondrial function due to aging and reduce the oxidative stress and inflammatory response of the ovary and oviduct (43), thus improving the reproductive pathway. Zhou et al. (44) found that the addition of antioxidants improved performance probably through enhancing the immunity and attenuating inflammation of laying hens. Here in this study, it was found that OFO induced an increase in serum inflammatory factors, and the dietary addition of LAM significantly increased immunoglobulin (IgA, IgG, and IgM) levels and reduced the inflammatory factors (TNF-α, IL-1β, IL-6, and IFN-γ). As a derivative of LA, these results suggested that LAM may also improve egg production performance by alleviating oxidative stress and inflammatory response.

Moreover, several studies found that egg-laying rates in the late egg-laying period have a positive correlation with serum estrogen levels (1, 45). Li et al. (46) found that the egg production performance was improved by dietary supplements with soya saponin *via* increasing serum estrogen level. It is concluded that the addition of antioxidants to the diet can improve egg production to some extent by improving estrogen secretion. Recent studies have reported that LA can improve estrogen secretion in rats caused by ovarian menopause (47) and oxidative damage (48). In the present study, LAM supplementation for old laying hens increased the levels of E2 and P in the serum significantly. Both E2 and P are the main estrogens produced by the ovaries, which promote the development of the reproductive system and enhance fertility in humans and animals. Therefore, it seems plausible that LAM might also improve egg production rate by increasing the secretion of E2 and P.

The amount of estrogen secretion is related to follicle development and maturation. Besides, it is well known that the egg-laying rate of hens depends mainly on the follicle formation and ovulation process (31), the number of follicles often represents egg production. In a comparative transcriptomic analysis of duck ovaries with different egg-laying rates, 25 differential genes associated with follicle development were identified. These differential genes were involved in multiple estrogen-related signaling pathways (49). Oxidative stress is considered to be a major factor in follicle development. Oxidative stress could trigger the apoptosis of most germ cells and even follicles in the ovary, and its presence in the follicular fluid decreases follicle quality and reduces reproductive outcomes. On the other hand, antioxidants reduced the levels of ROS and prevented oxidative stress-mediated germ cell apoptosis, thus reducing follicular depletion (50). Several *in vitro* experiments have found that certain concentrations of LA can promote the development and maturation of the preantral follicle, such as mouse and equine (51, 52). Our finding indicated that the OFO reduced the number of dominant follicles, while the addition of LAM to the diet alleviated the adverse effect of OFO on follicles. Therefore, LAM could resist the effects of oxidative stress on follicles and improve egg production.

In summary, this study provided evidence that OFO induces inflammation and oxidative damage to the oviduct in laying hens, thereby adversely affecting egg production performance. While the addition of LAM to the diet enhanced the egg-laying rate of hens during the late laying period by increasing the oviduct antioxidant capacity, serum immunity, estrogen levels and antioxidant capacity, and number of dominant follicles. On the basis of our work, we can surmise that LAM may be useful as a potential candidate against oxidative injury and a promising agent for improving the utilization of old laying hens.

## Data Availability Statement

The raw data supporting the conclusions of this article will be made available by the authors, without undue reservation.

## Ethics Statement

All animal protocols used in this study were approved and carried out according to the guidelines for the ethical treatment of animals by the Institutional Animal Care and Use Committee of China Agricultural University (Beijing, China; No. AW71202202-1-1).

## Author Contributions

QL, WL, JiatZ, LZ, CJ, JianZ, and QM designed the study. QL, WL, JiatZ, LZ, CJ, JianZ, SH, and QM conducted the experiments and draft the manuscript. QL, SH, and QM polished the manuscript and finished the submission. QL, WL, JianZ, CJ, and QM guided to analyses the experimental data. QL, SH, CJ, and QM helped with revisiting and reviewing the manuscript. All authors read and approved the final manuscript.

## Funding

This research was funded by the National Natural Science Foundation of China (Grant No. 31772621), a Special Fund for China Agricultural Research System Program (Grant No. CARS-40-K08), and the Special Fund from Chinese Universities Scientific Fund (Grant No. 2018TC043).

## Conflict of Interest

The authors declare that the research was conducted in the absence of any commercial or financial relationships that could be construed as a potential conflict of interest.

## Publisher's Note

All claims expressed in this article are solely those of the authors and do not necessarily represent those of their affiliated organizations, or those of the publisher, the editors and the reviewers. Any product that may be evaluated in this article, or claim that may be made by its manufacturer, is not guaranteed or endorsed by the publisher.
