# Supplementary material for: Lipoamide Alleviates Oxidized Fish Oil-Induced Host Inflammatory Response and Oxidative Damage in the Oviduct of Laying Hens
Source: Front Vet Sci. 2022 Apr 4;9:875769. doi: 10.3389/fvets.2022.875769 (PMC9040665; doi:10.3389/fvets.2022.875769)
Supplement: Supplementary file 1 [file Table_1.docx]

**Supplementary Table 1. Composition and nutrient levels of the basal diet (air-dry basis).**

| **Ingredients (%)** | **Composition** | **Nutrient levels (%)** | **Value** |
| --- | --- | --- | --- |
| Corn | 64.60 | Metabolic energy (Kcal/kg) | 2690 |
| Soybean meal | 24.20 | Crude Protein | 16.04 |
| calcium hydrophosphate | 1.70 | Calcium | 3.60 |
| Limestone | 8.20 | Available phosphorus (%) | 0.39 |
| Sodium chloride | 0.30 | Methionine | 0.38 |
| DL-methionine (98%) | 0.12 | Sulfur-containing amino acid | 0.65 |
| Choline chloride (50%) | 0.10 | Lysine | 0.78 |
| Vitamin premix^a^ | 0.04 | Threonine | 0.59 |
| Trace elements premix^b^ | 0.30 |  |  |
| Ethoxyquin (60%) | 0.02 |  |  |
| Zeolite powder + lipoamide | 0.42 |  |  |

^a^premix provided the following per kilogram of the diet: vitamin A, 11700 IU; vitamin D3, 3600IU; vitamin E, 21 IU; vitamin K_3_, 4.2 mg; vitamin B_1_, 3.0 mg; vitamin B_2_, 10.2 mg; folic acid, 0.9 mg; pantothenic acid calcium, 15.0 mg; niacin, 45.0 mg; vitamin B_6_, 5.4 mg; vitamin B_12_, 24 μg; biotin, 0.15 mg.

^b^premix provided the following per kilogram of the diet: Cu (as copper sulfate) 6.00 mg; Fe (as ferrous sulfate) 60.0 mg; Mn (as manganese sulfate) 80.0 mg; Zn (as zinc sulfate) 83.0 mg; Se (as sodium selenite) 0.30 mg; I (as potassium iodide) 1.00 mg.
